# A New Insight for the Identification of Oncogenic Variants in Breast and Prostate Cancers in Diverse Human Populations, With a Focus on Latinos

**DOI:** 10.3389/fphar.2021.630658

**Published:** 2021-04-12

**Authors:** Nelson M. Varela, Patricia Guevara-Ramírez, Cristian Acevedo, Tomás Zambrano, Isaac Armendáriz-Castillo, Santiago Guerrero, Luis A. Quiñones, Andrés López-Cortés

**Affiliations:** ^1^ Laboratory of Chemical Carcinogenesis and Pharmacogenetics, Department of Basic and Clinical Oncology, Faculty of Medicine, University of Chile, Santiago, Chile; ^2^ Latin American Network for the Implementation and Validation of Clinical Pharmacogenomics Guidelines (RELIVAF-CYTED), Madrid, Spain; ^3^ Department of Basic and Clinical Oncology, Clinical Hospital University of Chile, Santiago, Chile; ^4^ Department of Medical Technology, Faculty of Medicine, University of Chile, Santiago, Chile; ^5^ Centro de Investigación Genética y Genómica, Facultad de Ciencias de la Salud Eugenio Espejo, Universidad UTE, Quito, Ecuador; ^6^ Department of Computer Science and Information Technologies, Faculty of Computer Science, University of A Coruna, A Coruña, Spain

**Keywords:** breast, prostate, oncogenic variants, latino population, precision oncology

## Abstract

**Background:** Breast cancer (BRCA) and prostate cancer (PRCA) are the most commonly diagnosed cancer types in Latin American women and men, respectively. Although in recent years large-scale efforts from international consortia have focused on improving precision oncology, a better understanding of genomic features of BRCA and PRCA in developing regions and racial/ethnic minority populations is still required.

**Methods:** To fill in this gap, we performed integrated *in silico* analyses to elucidate oncogenic variants from BRCA and PRCA driver genes; to calculate their deleteriousness scores and allele frequencies from seven human populations worldwide, including Latinos; and to propose the most effective therapeutic strategies based on precision oncology.

**Results:** We analyzed 339,100 variants belonging to 99 BRCA and 82 PRCA driver genes and identified 18,512 and 15,648 known/predicted oncogenic variants, respectively. Regarding known oncogenic variants, we prioritized the most frequent and deleterious variants of BRCA (*n* = 230) and PRCA (*n* = 167) from Latino, African, Ashkenazi Jewish, East Asian, South Asian, European Finnish, and European non-Finnish populations, to incorporate them into pharmacogenomics testing. Lastly, we identified which oncogenic variants may shape the response to anti-cancer therapies, detailing the current status of pharmacogenomics guidelines and clinical trials involved in BRCA and PRCA cancer driver proteins.

**Conclusion:** It is imperative to unify efforts where developing countries might invest in obtaining databases of genomic profiles of their populations, and developed countries might incorporate racial/ethnic minority populations in future clinical trials and cancer researches with the overall objective of fomenting pharmacogenomics in clinical practice and public health policies.

## Introduction

Cancer is the second leading cause of death globally (Bray et al., 2018); meanwhile, breast cancer (BRCA) and prostate cancer (PRCA) are the most commonly diagnosed cancer types in Latin American women and men, respectively, (López-Cortés et al., 2017). BRCA and PRCA are complex and heterogeneous diseases characterized by an intricate interplay between different biological features, such as driver mutations, ethnicity, protein expression deregulation, signaling pathway alterations, and environmental determinants (López-Cortés et al., 2013; López-Cortés et al.,2018; López-Cortés et al.,2020b; López-Cortés et al.,2020a).

Starting with the Human Genome Project in 1990, genomics has progressively become an essential tool in basic and translational research (Green et al., 2020). The development of high-throughput technologies focused on large-scale DNA sequencing has allowed us to better understand the molecular landscape of oncogenesis. Thus, considerable progress has been made in discovering cancer driver genes (Kandoth et al., 2013; Lawrence et al., 2014), coding and non-coding cancer driver mutations (Sjöblom et al., 2006; Tamborero et al., 2013; Porta-Pardo et al., 2017; Rheinbay et al., 2020), germline variants (Lu et al., 2015), druggable enzymes (Rubio-Perez et al., 2015), and drug resistance (Vasan et al., 2019).

A main goal in oncology research is to understand the mechanisms of malignant cell transformation to develop efficient therapeutic approaches. One milestone towards this objective is the identification of cancer driver genes carrying mutations capable of driving BRCA and PRCA tumorigenesis. Nowadays, it is known that cancer driver genes are under positive selection in tumorigenesis, and the development of carefully designed bioinformatics pipelines such as the Integrative OncoGenomics (IntOGen) and the Cancer Genome Interpreter (CGI) is fundamental to identify oncogenic variants across tumors (Tamborero et al., 2018; Martínez-Jiménez et al., 2020). Similarly, The Cancer Genome Atlas (TCGA) and the Therapeutically Applicable Research to Generate Effective Treatments (TARGET) projects have established molecular tumor classification based on DNA, RNA and protein alterations (ICGC/TCGA Pan-Cancer Analysis of Whole Genomes Consortium, 2020).

These genomic signatures are allowing the development of personalized cancer treatments. Over the past years it has become clear that oncological patients, diagnosed with the same cancer type, may have different responses to generic treatments such as radiation or chemotherapy. To overcome these variable responses, cancer precision medicine aims to provide the right dose of the right drug for the right patient at the right time (Quinones et al., 2014). Thus, precision medicine has become an important tool in cancer treatment; it allows the identification of specific mutations in driver genes responsible for tumor progression (ICGC/TCGA Pan-Cancer Analysis of Whole Genomes Consortium, 2020). Based on each human multi-omics profile, drug development can be tailored for each individual improving not only efficiency of the drug but minimizing the possibility of acquiring adverse reactions (López-Cortés et al., 2020c).

Despite these efforts, fundamental and applied cancer researchers have failed to include ethnically diverse populations (Guerrero et al., 2018). In that respect, several studies have shown that race/ethnicity has a great impact on cancer incidence, survival, drug response, molecular pathways, and epigenetics (Ma et al., 2010; Patel, 2015; Kader and Ghai, 2017). Astonishingly, relevant cancer genomic databases, such as TCGA and TARGET, are overrepresented by Caucasian individuals (91.1%) (Guerrero et al., 2018). Consequently, this bias greatly limits the development of pharmacogenomics (PGx) and precision medicine in developing regions, such as Latin America. To fill in this gap, we performed integrated *in silico* analyses to elucidate oncogenic variants from BRCA and PRCA driver genes, and to calculate their deleteriousness scores and allele frequencies from seven human populations worldwide, with a focus on the Latino population.

## Methods

### Incidence and Mortality of BRCA and PRCA

The Global Cancer Observatory (GLOBOCAN) (https://gco.iarc.fr/) enables a comprehensive assessment of the cancer burden worldwide (Bray et al., 2018). From the latest version of GLOBOCAN 2020, we have retrieved the estimated crude incidence and mortality rates related to the top cancer types worldwide, and the estimated crude incidence and mortality rates of BRCA and PRCA from Latin American and the Caribbean countries.

### BRCA and PRCA Driver Genes

The intOGen (https://www.intogen.org) framework identifies cancer genes and pinpoints their mechanism of action across tumor types (Martínez-Jiménez et al., 2020). The current version of the intOGen pipeline uses seven methods to identify cancer driver genes from somatic point mutations: dNdScv (Martincorena et al., 2017), CBaSE (Weghorn and Sunyaev, 2017), MutPanning (Dietlein et al., 2020), OncodriveCLUSTL (Arnedo-Pac et al., 2019), HotMAPS (Tokheim et al., 2016), smRegions (Martínez-Jiménez et al., 2020), and OncodriveFML (Mularoni et al., 2016). Therefore, we have retrieved 99 BRCA driver genes and 82 PRCA driver genes from intOGen, and have identified its involvement as oncogenes (Sondka et al., 2018), tumor suppressor genes (Sondka et al., 2018), kinase genes (Manning et al., 2002), DNA-repair genes (Chae et al., 2016), RNA-binding proteins (Hentze et al., 2018), cell cycle genes (Bar-Joseph et al., 2008), and cancer immunotherapy genes (Patel et al., 2017). Lastly, BRCA and PRCA driver gene sets are fully detailed in Supplementary Table S1.

### Identification of Oncogenic Variants

The identification of oncogenic variants was divided in two steps. In the first step, we extracted 339,100 single nucleotide variants and insertion/deletion variants belonging to 99 BRCA diver genes (*n* = 183,616) and 82 PRCA driver genes (*n* = 155,484) from the Genome Aggregation database (gnomAD v2.2.1), using GRCh37/hg19 as the human genome reference (Collins et al., 2020; Karczewski et al., 2020). In the second step, we performed the OncodriveMUT method integrated into the Cancer Genome Interpreter platform (https://www.cancergenomeinterpreter.org) to assess the tumorigenic potential of the 339,100 aforementioned genomic variants (Tamborero et al., 2018). OncodriveMUT is a developed rule-based approach that combines genomic features such as gene signals of positive selection, clusters of somatic mutations, gene mechanism of action, and regions depleted by germline variants to classify driver mutations into known, predicted tier 1, predicted tier 2, and passenger mutations using the Catalog of Validated Oncogenic Mutations (Tamborero et al., 2018).

### Deleteriousness Score of Oncogenic Variants

Combined Annotation-Dependent Depletion (CADD) (https://cadd.gs.washington.edu/) is an integrative annotation built from more than 60 genomic features, and measures the deleteriousness of single nucleotide variants as well as insertion/deletion variants in the human genome (Kircher et al., 2014; Rentzsch et al., 2019). In this study, we calculated the CADD phred score for ranking the deleteriousness of known and predicted oncogenic variants located in BRCA and PRCA driver genes. The deleteriousness of oncogenic variants was categorized according to its CADD phred score in very high (30–50), high (25–30), medium (15–25), low (10–15), and very low (0–10).

### Allele Frequencies in Human Populations

The gnomAD (https://gnomad.broadinstitute.org/) is a database that harmonize exome and genome sequencing data from a variety of large-scale sequencing projects worldwide (Karczewski et al., 2020). The gnomAD database version 2.1.1 is integrated by 15,708 exomes and 125,748 genomes (total = 141,456). In this study, we calculated the allele frequencies of BRCA and PRCA oncogenic variants belonging to seven human populations, such as Latino (424 exomes and 17,296 genome), African (4,359 exomes and 8,128 genomes), Ashkenazi Jewish (145 exomes and 5,040 genomes), East Asian (780 exomes and 9,197 genomes), South Asian (15,308 genomes), European Finnish (1,738 exomes and 10,824 genomes), and European non-Finnish (7,718 exomes and 56,885 genomes) (Collins et al., 2020; Karczewski et al., 2020; Wang et al., 2020).

### Validation of Known Oncogenic Variants Through the Pan-Cancer Atlas

The Pan-Cancer Atlas project, which belongs to TCGA consortium, provides a comprehensive, in-depth, and interconnected understanding of human cancer, and is an essential resource for the development of new treatments in the pursuit of precision medicine (Hoadley et al., 2018; Huang et al., 2018). Therefore, the previously obtained known oncogenic variants were identified and the allele frequencies were calculated in a cohort of 850 TCGA-BRCA patients encompassing 162 black/African individuals, 600 white individuals (not Hispanic or Latino), 29 white individuals (Hispanic or Latino), and 59 Asian individuals; and in a cohort of 150 TCGA-PRAD patients encompassing seven black/African individuals, and 143 white individuals (not Hispanic or Latino). Lastly, mutational data was taken from the Genomics Data Commons of the National Cancer Institute (https://portal.gdc.cancer.gov/), and the cBioPortal database (http://www.cbioportal.org/) (Cerami et al., 2012; Gao et al., 2013).

### Current Pharmacogenomics Guidelines in Clinical Practice

PharmGKB (https://www.pharmgkb.org/) is a pharmacogenomics knowledge resource that encompasses potentially clinically actionable gene-drug associations and precise guidelines for the application of pharmacogenomics in clinical practice (Whirl-Carrillo et al., 2012; Barbarino et al., 2018). This database collects information from the Clinical Pharmacogenetics Implementation Consortium (Saito et al., 2016; Relling et al., 2020), the Canadian Pharmacogenomics Network for Drug Safety (Ross et al., 2010), the Royal Dutch Association for the Advancement of Pharmacy (Swen et al., 2011), the National Comprehensive Cancer Network (NCCN), and the European Society for Medical Oncology (ESMO). Consequently, we have retrieved clinical annotations, gene-drug pairs, and genomic variants associated to BRCA and PRCA pharmacogenomics guidelines.

### *In silico* Drug Prescription

Another CGI approach is the *in silico* drug prescription that contains the putative biomarker of drug response found in the tumor organized according to distinct levels of clinical relevance. The CGI employs two resources to explore the association between genomic variants and drug response: the Cancer Biomarker database (Dienstmann et al., 2015), and the Cancer Bioactivities database (Tamborero et al., 2018). Therefore, we performed an in silico analysis to determine the druggability of known and predicted oncogenic variants located in BRCA and PRCA driver genes, and consequently the most relevant precision oncology treatments.

### Clinical Trials

The Open Targets Platform (https://www.targetvalidation.org) is comprehensive and robust data integration for access to and visualization of drugs involved in clinical trials associated with BRCA and PRCA proteins, detailing its phase, type, action type, and target class (Carvalho-Silva et al., 2019). Additionally, we created Sankey plots to better understand which drugs are involved in the most advanced phases (3 and 4) of clinical trials in both cancer types.

## Results

### Incidence and Mortality of BRCA and PRCA

**FIGURE 1:**
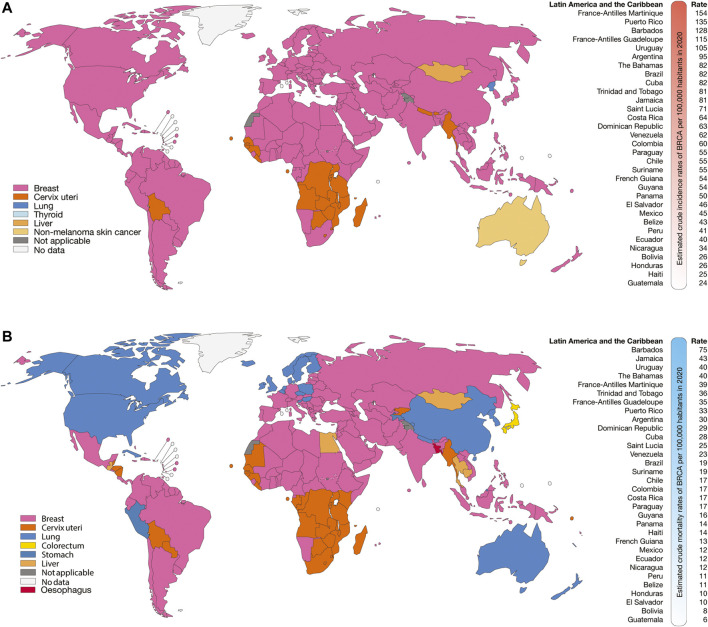
Epidemiology of BRCA. **(A)** Top cancer crude incidence per country in females of all ages **(left)**, and ranking of estimated crude incidence rates of BRCA per 100,000 inhabitants in Latin American and the Caribbean countries **(right)**. **(B)** Top cancer mortality per country in females of all ages **(left)**, and ranking of mortality crude rates of BRCA per 100,000 inhabitants in Latin American and the Caribbean countries **(right)**.

**FIGURE 2:**
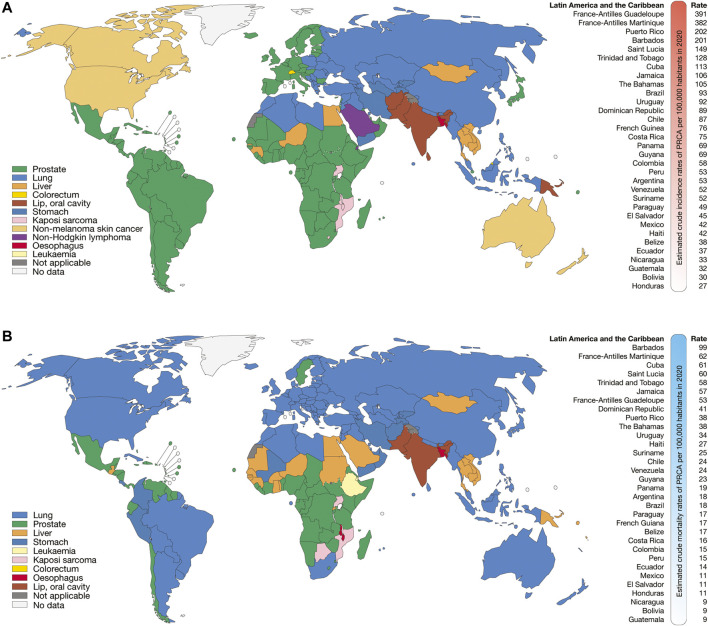
Epidemiology of PRCA. **(A)** Top cancer crude incidence per country in males of all ages **(left)**, and ranking of estimated crude incidence rates of PRCA per 100,000 inhabitants in Latin American and the Caribbean countries **(right)**. **(B)** Top cancer mortality per country in males of all ages **(left)**, and ranking of mortality crude rates of PRCA per 100,000 inhabitants in Latin American and the Caribbean countries **(right)**.

According to GLOBOCAN 2020, the Latin American and the Caribbean countries with the highest estimated crude incidence rates of BRCA per 100,000 inhabitants were France-Antilles Martinique (154), Puerto Rico (135), Barbados (128), France-Antilles Guadeloupe (115), Uruguay (105), Argentina (95), The Bahamas (82), and Brazil (82) (Figure 1A); the countries with the highest estimated crude mortality rates of BRCA were Barbados (75), Jamaica (43), Uruguay (40), The Bahamas (40), France-Antilles Martinique (39), Trinidad and Tobago (36), and France-Antilles Guadeloupe (35) (Figure 1B); the countries with the highest estimated crude incidence rates of PRCA were France-Antilles Guadeloupe (391), France-Antilles Martinique (382), Puerto Rico (202), Barbados (201), Saint Lucia (149), Trinidad and Tobago (128), Cuba (113), and Jamaica (106) (Figure 2A); lastly, the countries with the highest estimated crude mortality rates of PRCA were Barbados (99), France-Antilles Martinique (62), Cuba (61), Saint Lucia (60), Trinidad and Tobago (58), Jamaica (57), France-Antilles Guadeloupe (53), and Dominican Republic (41) (Figure 2B) (Bray et al., 2018).

### BRCA and PRCA Driver Genes

We have retrieved 99 BRCA driver genes and 82 PRCA driver genes from the intOGen framework (Martínez-Jiménez et al., 2020). Regarding BRCA driver genes, 41.4% were tumor suppressor genes (Sondka et al., 2018), 28.3% were oncogenes (Sondka et al., 2018), 15.2% were kinase genes (Manning et al., 2002), 10.1% encode RNA-binding proteins (Hentze et al., 2018), 9.1% were cancer immunotherapy genes (Patel et al., 2017), 4% were cell cycle genes (Bar-Joseph et al., 2008), and 4% were DNA repair genes (Chae et al., 2016). Regarding PRCA driver genes, 37.8% were tumor suppressor genes (Sondka et al., 2018), 31.7% were oncogenes (Sondka et al., 2018), 13.4% were cancer immunotherapy genes (Patel et al., 2017), 12.2% were kinase genes (Manning et al., 2002), 8.5% encode RNA-binding proteins (Hentze et al., 2018), 3.7% were cell cycle genes (Bar-Joseph et al., 2008), and 3.7% were DNA repair genes (Chae et al., 2016) (Supplementary Table 1).

### Identification of Oncogenic Variants

**FIGURE 3:**
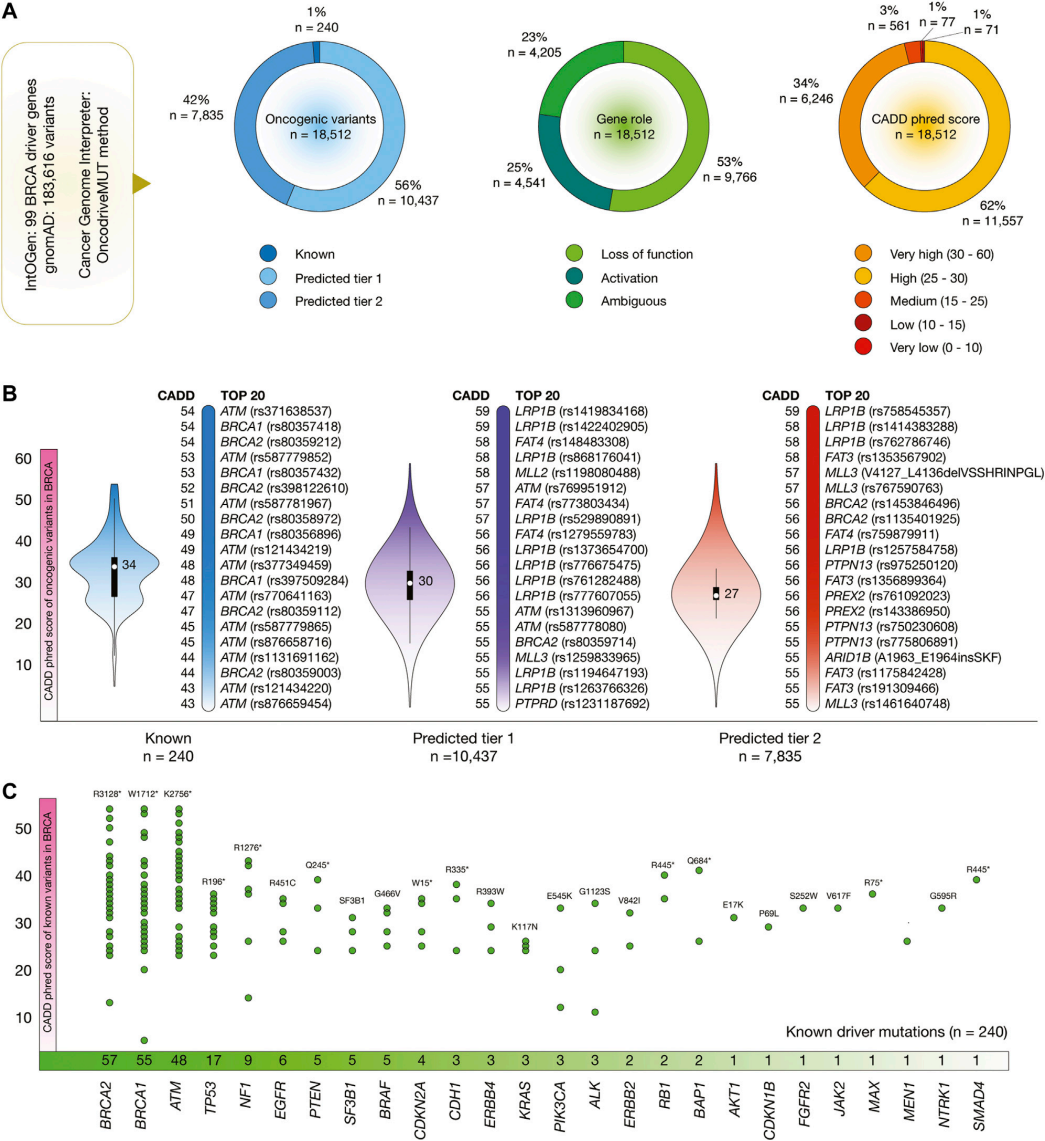
BRCA driver genes, oncogenic variants, and deleteriousness scores. **(A)** Features of BRCA driver genes and oncogenic variants. **(B)** Bean plots of CADD phred scores of BRCA oncogenic variants, and ranking of known oncogenic variants with the highest CADD phred scores. **(C)** BRCA driver genes with the highest number of oncogenic variants.

Figure 3 shows the results of the OncodriveMUT analysis to identify oncogenic variants in 99 BRCA driver genes. After the analysis of 183,616 variants, we identified 18,512 oncogenic variants. Of them, 240 (1%) were known, 10,437 (56%) were predicted tier 1, and 7,835 (42%) were predicted tier 2. Regarding gene role, 9,766 (53%) variants produced protein loss of function and 4,541 (25%) produced protein activation. In addition, 6,246 (34%) oncogenic variants had very high CADD phred score, and 11,557 (62%) had high score (Figure 3A).

Figure 3B shows violin plots and ranking of CADD phred score of known, predicted tier 1 and tier 2 oncogenic variants in BRCA. The known oncogenic variant with the highest score was *ATM* rs371638537 (score = 54). The predicted tier 1 variant with the highest score was *LRP1B* rs1419834168 (59), and the predicted tier 2 variant with the highest score was *LRP1B* rs758545357 (54). The ranking of the 18,512 BRCA oncogenic variants is fully detailed in the Supplementary Table S2.

Figure 3C details the number of known oncogenic variants per BRCA driver gene. Genes with the highest number of known variants were *BRCA2* (*n* = 57), *BRCA1* (55), *ATM* (48), *TP53* (17), *NF1* (9), *EGFR* (6), *PTEN* (5), *SF3B1* (5), *BRAF* (5), and *CDKN2A* (4).

**FIGURE 4:**
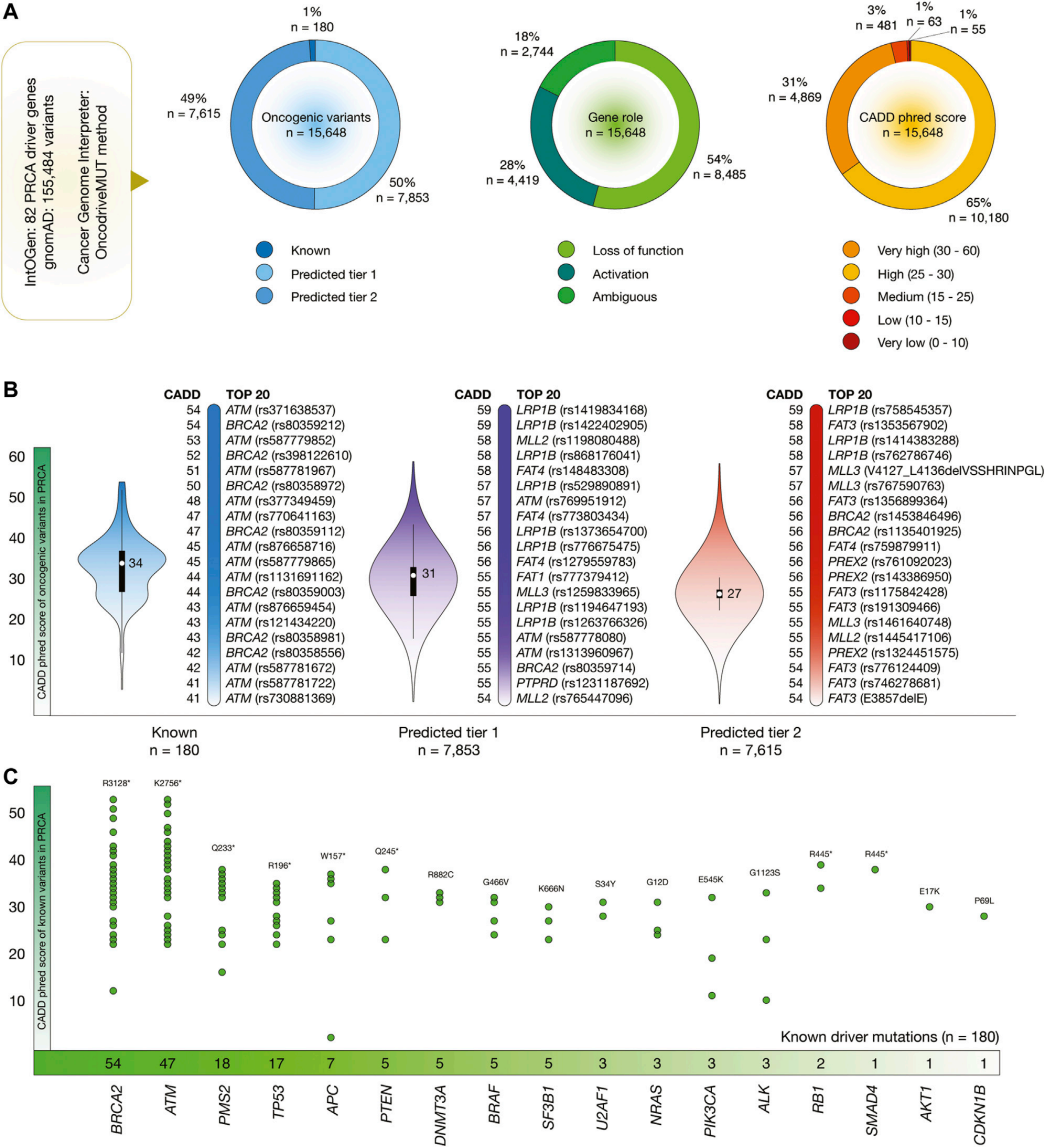
PRCA driver genes, oncogenic variants, and deleteriousness scores. **(A)** Features of PRCA driver genes and oncogenic variants. **(B)** Bean plots of CADD phred scores of PRCA oncogenic variants, and ranking of known oncogenic variants with the highest CADD phred scores. **(C)** PRCA driver genes with the highest number of oncogenic variants.

Figure 4 shows the results of the OncodriveMUT analysis to identify oncogenic variants in 82 PRCA driver genes. After the analysis of 155,484 variants, we identified 15,648 oncogenic variants. Of them, 180 (1%) were known, 7,853 (50%) were predicted tier 1, and 7,615 (49%) were predicted tier 2 oncogenic variants. Regarding gene role, 8,485 (54%) variants produced protein loss of function and 4,419 (28%) variants produced protein activation. Additionally, 4,869 (31%) oncogenic variants had very high CADD phred score, and 10,180 (65%) variants had high (Figure 4A).

Figure 4B shows violin plots and ranking of CADD phred score of known, predicted tier 1 and tier 2 oncogenic variants in PRCA. The known oncogenic variant with the highest score was *ATM* rs371638537 (score = 54), the predicted tier 1 variant with the highest score was *LRP1B* rs1419834168 (59), and the predicted tier 2 variants with the highest score was *LRP1B* rs758545357 (59). The ranking of the 15,648 PRCA oncogenic variants is fully detailed in the Supplementary Table S3.

Finally, Figure 4C details the number of known oncogenic variants per PRCA driver gene. Genes with the highest number of known variants were *BRCA2* (*n* = 54), *ATM* (47), *PMS2* (18), *TP53* (17), *APC* (7), *PTEN* (5), *DNMT3A* (5), *BRAF* (5), *SF3B1* (5), and *U2AF1* (3).

### Deleteriousness Scores, Allele Frequencies, and Validation of Oncogenic Variants per Human Population

**FIGURE 5:**
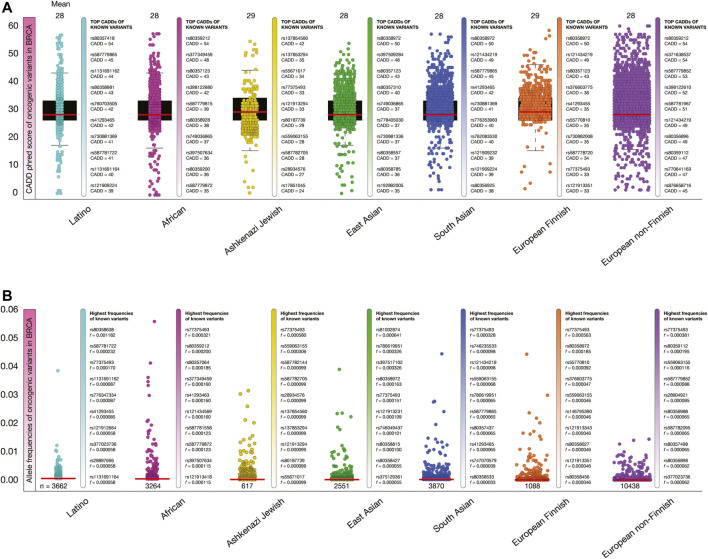
Deleteriousness scores and allele frequencies of BRCA oncogenic variants per human population. **(A)** Box plots show CADD phred scores of BRCA known and predicted oncogenic variants per human population. Vertical bars show ranking of known oncogenic variants with the highest CADD phred scores per human population. Red lines show the mean of deleteriousness scores per human population. **(B)** Box plots show allele frequencies of known and predicted BRCA oncogenic variants per human population. Vertical bars show ranking of known oncogenic variants with the highest allele frequencies per human population. Red lines show the mean of deleteriousness scores per human population. **(C)** Ranking of known oncogenic variants with the highest allele frequencies identified in breast cancer patients from TCGA-BRCA project.

Figure 5A shows box plots of CADD phred score of BRCA oncogenic variants per human population. The mean of deleteriousness score was 29 in Ashkenazi Jewish and South Asian populations, and 28 in Latino, African, East Asian, European Finnish and European non-Finish populations. The known oncogenic variant with the highest CADD phred score in Latinos was *BRCA1* rs80357418 (score = 54), in Africans was *BRCA2* rs80359212 (54), in Ashkenazi Jewish was *NF1* rs137854560 (42), in East Asians was *BRCA2* rs80358972 (50), in South Asians was *BRCA2* rs80358972 (50), in European Finnish was *BRCA2* 80358972 (50), and in European non-Finnish was *BRCA2* rs803559212 (54).

Figure 5B shows box plots of allele frequencies of oncogenic variants in BRCA driver genes per human population. The known oncogenic variant with the highest allele frequency in Latinos was *BRCA2* rs80358638 (*f* = 0.001182), in Africans was *JAK2* rs77375493 (0.000321), in Ashkenazi Jewish was *JAK2* rs77375493 (0.000580), in East Asians was *ERBB4* rs535202189 (0.000870), in South Asians was *JAK2* rs77375493 (0.000328), in European Finnish was *JAK2* rs77375493 (0.000563), and in European non-Finnish was *JAK2* rs77375493 (0.000381). The allele frequencies of the 18,512 BRCA oncogenic variants are fully detailed in the Supplementary Table S4.

Additionally, we identified some of the previously obtained allele frequencies of known oncogenic variants using the TCGA-BRCA project. From the 240 known oncogenic variants, we identified 32 variants and calculated its allele frequencies in 850 TCGA-BRCA patients with ethnicity data. The known oncogenic variants with the highest allele frequencies in the 162 black/African individuals were *PIK3CA* H1047R (*f* = 0.068), *AKT1* E17K (0.037), *PIK3CA* E542K (0.037), *TP53* R175H (0.037), and *TP53* Y220C (0.019); in the 600 white individuals (not Hispanic or Latino) were *PIK3CA* H1047R (0.419), *PIK3CA* E542 (0.154), *AKT1* E17K (0.093), *TP53* R175H (0.056), and *PIK3CA* H1047L (0.056); in the 29 white individuals (Hispanic or Latino) were *PIK3CA* H1047R (0.019), *PIK3CA* E542K (0.006), *PIK3CA* H1047L (0.006), *TP53* R273H (0.006), and *CDH1* Q23* (0.006); and in the 59 Asian individuals were *PIK3CA* H1047R (0.068), *PIK3CA* E542K (0.037), *TP53* R273H (0.012), *AKT1* E17K (0.037), and *TP53* R175H (0.037) (Supplementary Table S5).

**FIGURE 6:**
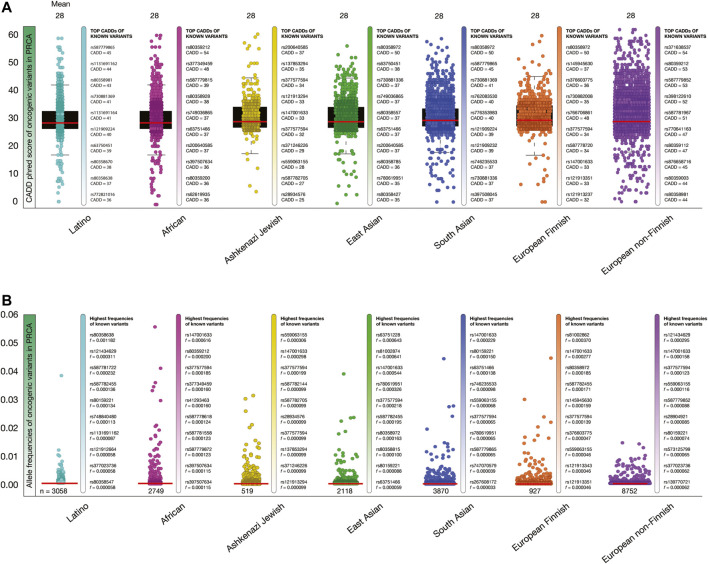
Deleteriousness scores and allele frequencies of PRCA oncogenic variants per human population. **(A)** Box plots show CADD phred scores of PRCA known and predicted oncogenic variants per human population. Vertical bars show ranking of known oncogenic variants with the highest CADD phred scores per human population. Red lines show the mean of deleteriousness scores per human population. **(B)** Box plots show allele frequencies of known and predicted PRCA oncogenic variants per human population. Vertical bars show ranking of known oncogenic variants with the highest allele frequencies per human population. Red lines show the mean of deleteriousness scores per human population. **(C)** Ranking of known oncogenic variants with the highest allele frequencies identified in prostate cancer patients from TCGA-PRCA project.

Figure 6A shows box plots of CADD phred score of PRCA oncogenic variants per human population. The mean of deleteriousness score was 28 in the seven human populations. The known oncogenic variant with the highest CADD phred score in Latinos was *ATM* rs587779865 (score = 45), in Africans was *BRCA2* rs80359212 (54), in Ashkenazi Jewish was *PMS2* rs200640585 (37), in East Asians was *BRCA2* rs80358972 (50), in South Asians was *BRCA2* rs80358972 (50), in European Finnish was *BRCA2* rs80358972 (50), and in European non-Finnish was *ATM* rs371638537 (54).

Figure 6B shows box plots of allele frequencies of oncogenic variants in PRCA driver genes per human population. The known oncogenic variant with the highest allele frequency in Latinos was *BRCA2* rs80358638 (*f* = 0.001182), in Africans was *DNMT3A* rs147001633 (0.000616), in Ashkenazi Jewish was *SF2B1* rs559063155 (0.000306), in East Asians was *PMS2* rs63751228 (0.000643), in South Asians was *DNMT3A* rs147001633 (0.000229), in European Finnish was *BRCA2* rs81002862 (0.000370), and in European non-Finnish was *PMS2* rs121434629 (0.000295). The allele frequencies of the 15,648 PRCA oncogenic variants are fully detailed in the Supplementary Table S6.

Lastly, we identified some of the previously obtained allele frequencies of known oncogenic variants using the TCGA-PRAD project. From the 180 known oncogenic variants, we identified five variants and calculated its allele frequencies in 150 TCGA-PRAD patients. The known oncogenic variant with the highest allele frequencies in the seven black/African individuals was *TP53* R158H (*f* = 0.142); and in the 143 white individuals (not Hispanic or Latino) were AKT1 E17K (0.007), TP53 R175H (0.007), TP53 R282W (0.007), TP53 R248Q (0.007), and TP53 R158H (0.007) (Supplementary Table S7).

### Current Pharmacogenomics Guidelines in Clinical Practice

**FIGURE 7:**
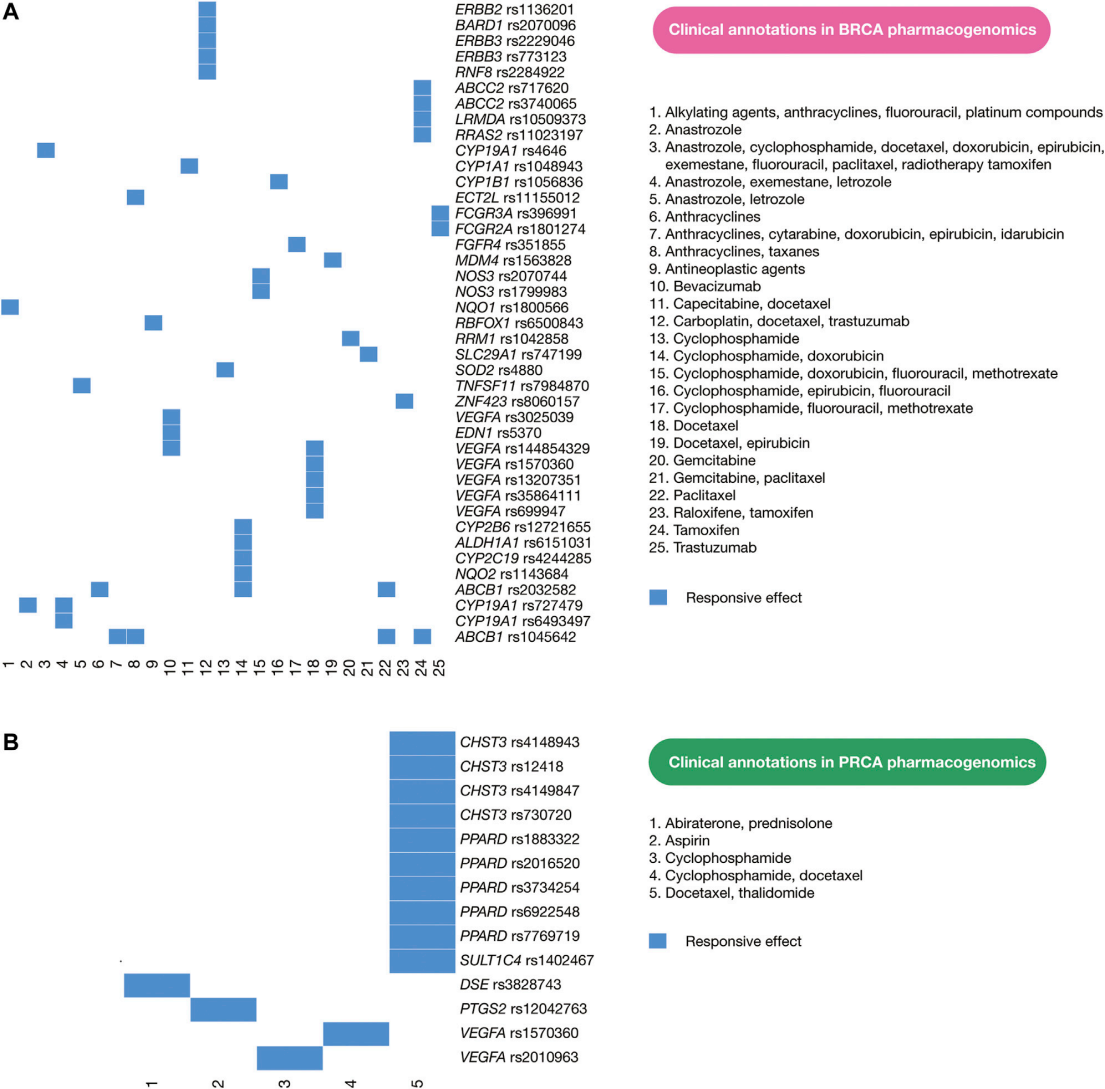
Current pharmacogenomics guidelines. **(A)** Clinical annotations of BRCA pharmacogenomics focused on responsive drug effects on human proteins. **(B)** Clinical annotations of PRCA pharmacogenomics focused on responsive drug effects on human proteins. These clinical annotations encompass cancer driver proteins and non-cancer driver proteins.

PharmGKB details the current status of pharmacogenomics guidelines applied in clinical practice of patients with BRCA and PRCA. Clinical annotations provide information about variant-drug pairs based on variant annotations (Whirl-Carrillo et al., 2012; Barbarino et al., 2018). Regarding BRCA, there are currently 160 clinical annotations with responsive and resistant effects involving 73 human genes. Of them, 47 clinical annotations have responsive drug effects on 30 human proteins as shown in Figure 7A, and 12 clinical annotations have responsive and resistant drug effects on BRCA driver genes. For instance, carboplatin, docetaxel, and trastuzumab have efficacy on patients with *ERBB3* rs773123, *ERBB3* rs2229046, and *ERBB2* rs1136201; docetaxel and epirubicin have efficacy on patients with *MDM4* rs1563828; exemestane generates toxicity on patients with *ESR1* rs2813543; everolimus produces toxicity on patients with *PIK3R1* rs10515074; tamoxifen generates toxicity on patients with *NCOA1* rs1804645; cyclophosphamide, doxorubicin, and fluorouracil produces toxicity on patients with *ATM* rs1801516; examestane and letrozole generates toxicity on patients with *ESR1* rs9322335; letrozole produces toxicity on patients with *ERS1* rs4870061; cyclophosphamide, epirubicin, and fluorouracil generates toxicity on patients with *TP53* rs4968187; and trastuzumab produces toxicity on patients with *ERBB2* rs1136201 (Supplementary Table S8). Regarding PRCA, there are currently 33 clinical annotations with responsive and resistant drug effects involving 15 human genes. Of them, 14 clinical annotations have responsive drug effects on six human proteins as shown in Figure 7B, but no clinical association is related to PRCA driver genes (Supplementary Table S9). However, the identification of numerous oncogenic variants with high deleteriousness scores in BRCA and PRCA driver genes provides the ability to improve drug discovery on potential therapeutic targets.

### *In silico* Drug Prescription

**FIGURE 8:**
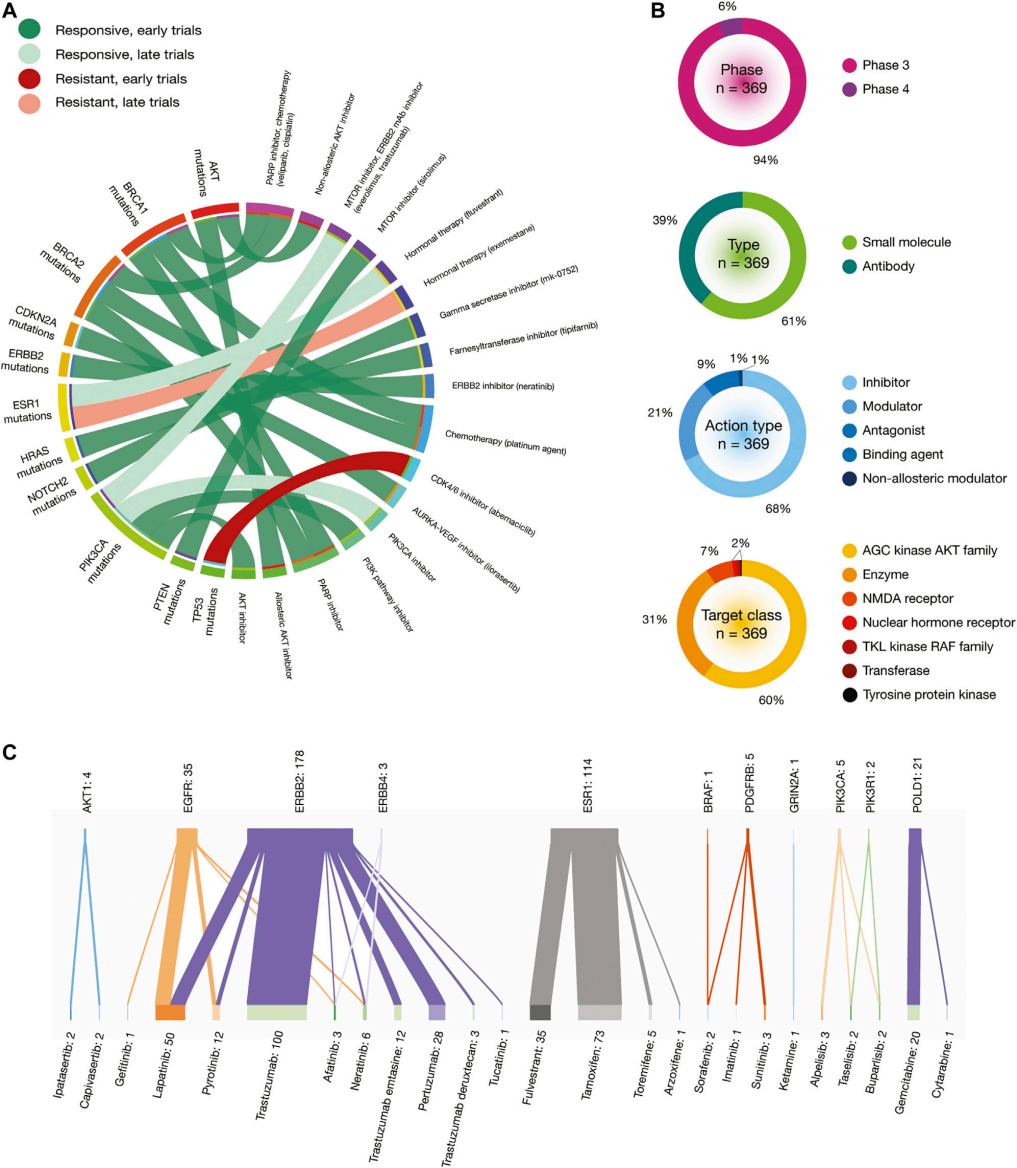
*In silico* drug prescriptions and clinical trials involved in BRCA. **(A)** Circos plot showing precision medicine application between drugs and BRCA driver mutations. **(B)** Clinical trial features on BRCA. **(C)** Sankey plot of drugs with the highest number of clinical trials on BRCA driver proteins.

One impressive resource that the CGI employs is the Cancer Biomarker database, an extension of a previous collection of genomic biomarkers of anti-cancer drug response, which contains 310 drugs across 130 cancer types (Dienstmann et al., 2015). Figure 8A shows a circos plot of putative biomarkers of drug response involved in BRCA treatments. Individuals with *AKT* oncogenic mutations have responsive treatments with non-allosteric and allosteric AKT inhibitors; *BRCA1* and *BRCA2* oncogenic mutations with PARP inhibitor (veliparib) and chemotherapy (cisplatin); *CDKN2A* oncogenic mutations with AURKA-VEGF inhibitor (ilorasertib); *ERBB2* oncogenic mutations with ERBB inhibitor (neratinib); *ESR1* oncogenic mutations with hormonal therapy (fluvestrant); *HRAS* oncogenic mutations with farnesyltransferase inhibitor (tipifarnib); *NOTCH2* oncogenic mutations with gamma secretase inhibitor (mk-0752); *PIK3CA* oncogenic mutations with MTOR inhibitor (everolimus) plus ERBB2 mAb inhibitor (trastuzumab), PIK3CA inhibitors, PI3K pathway inhibitors and AKT inhibitors; and *PTEN* oncogenic mutations with MTOR inhibitor (sirolimus). On the other hand, individuals with *TP53* oncogenic mutations have resistant treatment with CD4/6 inhibitor (abemaciclib); and *ESR1* oncogenic mutations with hormonal therapy (exemestane). All data is fully detailed in Supplementary Table S10.

**FIGURE 9:**
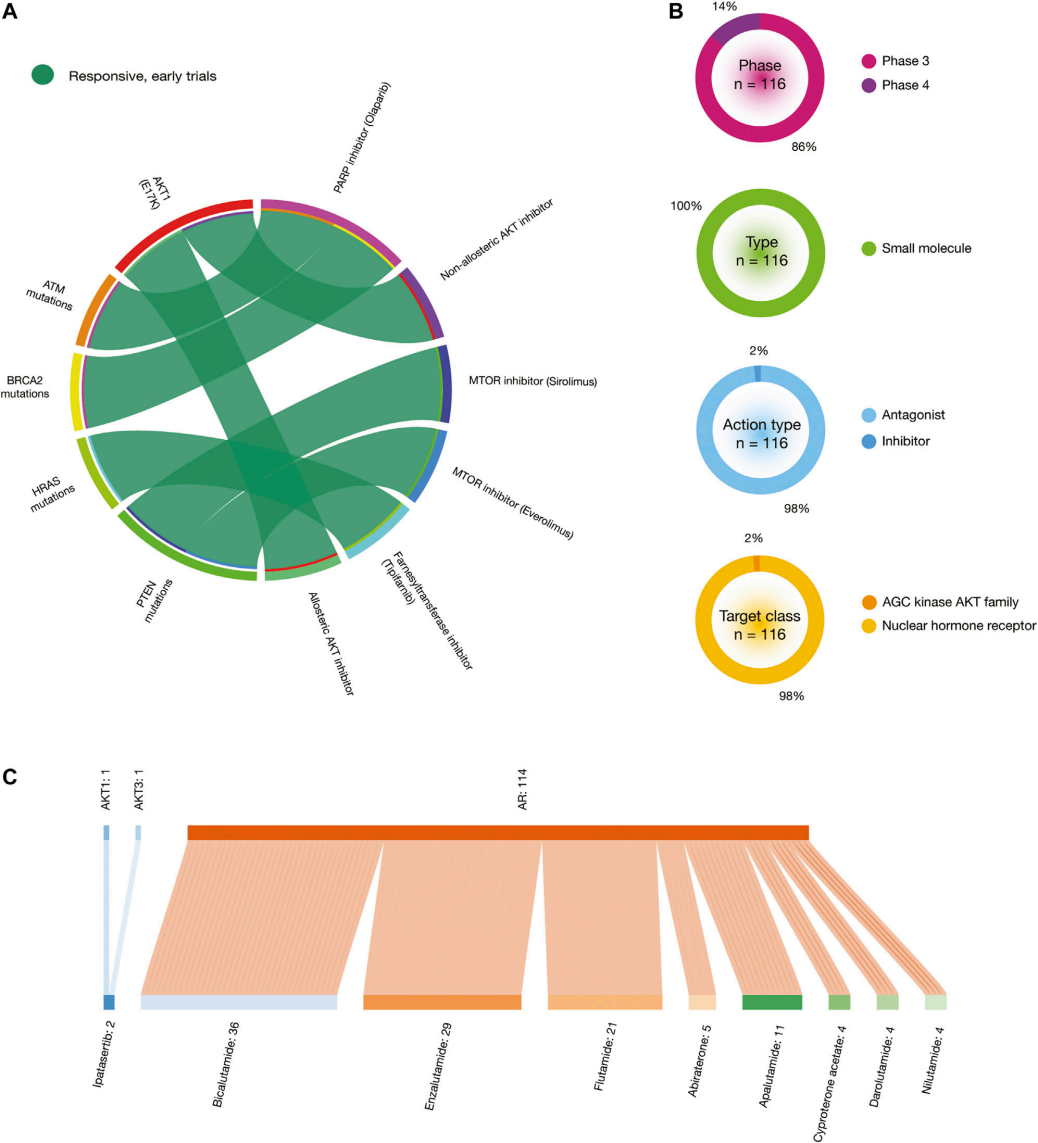
*In silico* drug prescriptions and clinical trials involved in PRCA. **(A)** Circos plot showing precision medicine application between drugs and PRCA driver mutations. **(B)** Clinical trial features on PRCA. **(C)** Sankey plots of drugs with the highest number of clinical trials on PRCA driver proteins.

Figure 9A shows a circos plot of putative biomarkers of drug response involved in PRCA treatments. Individuals with the *AKT1* E17K oncogenic mutation have responsive treatment with non-allosteric and allosteric AKT inhibitors; *ATM* oncogenic mutations with PARP inhibitor (olaparib); *BRCA2* oncogenic mutations with PARP inhibitor (olaparib); *HRAS* oncogenic mutations with farnesyltransferase inhibitor (tipifarnib); and *PTEN* oncogenic mutations with MTOR inhibitors (sirolimus and everolimus) (Figure 8A). All data is fully detailed in Supplementary Table S11.

### Clinical Trials

The Open Targets Platform shows the current status of clinical trials involved in BRCA and PRCA driver proteins (Carvalho-Silva et al., 2019). In regards to BRCA, there were 369 clinical trials in phase 3 (94%) and phase 4 (6%). Small molecules were the most analyzed type of drugs (61%), followed by antibodies (39%). Inhibitors were the most predominantly action type of drugs (68%), followed by modulators (21%), antagonists (9%), binding agents (1%), and non-allosteric modulators (1%). The target classes with the highest number of clinical trials was AGC kinase AKT family (60%), followed by enzymes (31%), NMDA receptors (7%), nuclear hormone receptors (0.5%), TKL kinase RAF family (0.5%), transferases (0.5%), and tyrosine protein kinases (0.5%) (Figure 8B). On the other hand, the Sankey plot showed 25 drugs currently analyzed in 369 clinical trials in 11 BRCA driver proteins. Druggable proteins with the highest number of clinical trials were ERBB2 (*n* = 178), ESR1 (*n* = 114), EGFR (*n* = 35), and POLD1 (*n* = 21). Lastly, drugs with the highest number of clinical trials in advanced stages were trastuzumab (*n* = 100), a recombinant humanized IgG1 monoclonal antibody against the ERBB2 receptor (Bange et al., 2001); tamoxifen (*n* = 73) that inhibits estrogen binding to its receptor (Jordan, 1993); lapatinib (*n* = 50) that is a 4-anilinoquinazoline kinase inhibitor of the intracellular tyrosine kinase domain of EGFR and ERBB2 (Xia et al., 2002); fulvestrant (*n* = 35) that achieves its anti-estrogen effects through downregulation and degradation of estrogen receptors (Chen et al., 2002); and pertuzumab (*n* = 28), a monoclonal antibody that targets the extracellular dimerization domain of ERBB2, thereby inhibiting intracellular signaling via the PI3K and MAP kinase pathways (Adams et al., 2006) (Figure 8C). All data is fully detailed in Supplementary Table S12.

Regarding PRCA, there are 116 clinical trials in phase 3 (86%) and phase 4 (14%). Small molecules were the only type of drugs analyzed (100%). Antagonists were the most predominantly action type of drugs (98%), followed by inhibitors (2%). The target classes with the highest number of clinical trials were AGC kinase AKT family (98%) and nuclear hormone receptors (2%) (Figure 9B). On the other hand, the Sankey plot showed nine drugs that are being analyzed in 116 clinical trials in 3 PRCA driver proteins. Druggable proteins with the highest number of clinical trials were AR (*n* = 114), AKT1 (*n* = 1), AKT3 (*n* = 1), and POLD1. Lastly, drugs with the highest number of clinical trials in advanced stages were bicalutamide (*n* = 36), a small molecule that blocks the action of androgens of adrenal and testicular origin (Chang et al., 1999); enzalutamide (*n* = 29), an androgen receptor inhibitor for the treatment of castration-resistant prostate cancer (Nadiminty et al., 2013); flutamide (*n* = 21), a nonsteroidal antiandrogen that blocks the action testosterone by binding to the androgen receptor (Balk, 2002); apalutamide (*n* = 11) that impairs the translocation of AR from the cytoplasm to the nucleus; and abiraterone (n = 5), a small molecule that is a derivative of steroidal progesterone and is an orally active inhibitor of CYP17A1 (de Bono et al., 2011) (Figure 9C). All data is fully detailed in Supplementary Table S13.

## Discussion

Precision oncology is a treatment paradigm that takes into account the molecular and cellular features of a tumor as well as its environment and additional traits of the individual, such as genetics and lifestyle, to create a tailor-made treatment (Le Tourneau et al., 2019). Most molecular alterations in tumors exist in multiple tumor types, and it has been hypothesized that anticancer therapy should be tailored to each patient according to their tumor molecular profile. Hence, the interpretation of molecular profiles through bioinformatics tools is imperative to analyze omics data and provide the most effective therapy to patients (Valencia and Hidalgo, 2012).

The most important aim in the interpretation of cancer genomes is to identify the variants responsible for tumorigenic traits. In this context, OncodriveMUT is a machine-learning approach integrated into the CGI platform to assess oncogenic variant’s tumorigenic potential. OncodriveMUT combines genomic features such as gene signals of positive selection, clusters of somatic mutations, gene mechanism of action, and regions depleted by germline variants (Tamborero et al., 2018). In this study, we analysed 183,616 variants located into 99 BRCA driver genes, and identified 18,512 known and predicted oncogenic variants. Of them, 240 were known oncogenic variants, and 9,766 were loss-of-function variants. Additionally, we analysed 155,484 variants located into 82 PRCA driver genes, and identified 15,648 known and predicted oncogenic variants. Of them, 180 were known oncogenic variants, and 8,485 were loss-of-function variants. Consequently, we calculated the CADD phred scores that represents the deleteriousness of single nucleotide variants as well as insertion/deletion variants involved in the molecular landscape of oncogenesis (Kircher et al., 2014; Rentzsch et al., 2019). The known BRCA oncogenic variants with the highest deleteriousness scores were *ATM* rs371638537 (CADD = 54), *BRCA1* rs80357418 (CADD = 54), and *BRCA2* rs80359212 (CADD = 54) (Figure 3B); and the known PRCA oncogenic variants with the highest deleteriousness scores were *ATM* rs371638537 (CADD = 54), and *BRCA2* rs80359212 (CADD = 54) (Figure 4B).

The ability to identify oncogenic variants and their deleteriousness scores in BRCA and PRCA tumors is an important step to apply PGx in clinical practice. Nevertheless, there are two main barriers for implementing PGx in developing regions. On the one hand, the most relevant cancer genome projects worldwide, such as TCGA(The Cancer Genome Atlas Research Network, 2013), TARGET or PCAWGC (ICGC/TCGA Pan-Cancer Analysis of Whole Genomes Consortium, 2020), are overrepresented by Caucasian individuals (91.1%), and do not include enough individuals from minority populations (Guerrero et al., 2018). On the other hand, developing regions lack of investment in cancer genomics tests, have fragmented healthcare systems, and have insufficient characterization of pharmacogenetics variability in their populations (Quinones et al., 2014). Therefore, in this study we proposed a new insight for identification of the most frequent oncogenic variants in the Latino, African, Ashkenazi Jewish, East Asian, South Asian, European Finnish, and European non-Finnish populations in order to focus economic resources on analyzing the most frequent and relevant molecular targets.

The gnomAD database harmonize exome and genome sequencing data from a variety of large-scale sequencing projects worldwide (Karczewski et al., 2020). We calculated allele frequencies of the previously identified known and predicted BRCA and PRCA oncogenic variants from Latinos, Africans, Ashkenazi Jewish, East Asians, South Asians, European Finnish, and European non-Finnish. Regarding BRCA, there are 42 known oncogenic variants with allele frequencies >0 in Latinos, 32 in Africans, 11 in Ashkenazi Jewish, 36 in East Asians, 35 in South Asians, 19 in European Finnish, and 156 in European non-Finnish (Supplementary Table S4). Regarding PRCA, there are 33 known oncogenic variants with allele frequencies >0 in Latinos, 27 in Africans, 12 in Ashkenazi Jewish, 25 in East Asians, 28 in South Asians, 15 in European Finnish, and 117 in European non-Finnish (Supplementary Table S5). Nevertheless, not all proteins carrying these oncogenic variants are actionable therapeutic targets or have clinical annotations in PGx guidelines.

Consequently, the second major aim of the effort to interpret cancer genomes is to identify which oncogenic variants may shape the response to anti-cancer therapies. After identifying the most frequent oncogenic variants per human population, we integrated these results with the current clinical annotations of the PGx guidelines from PharmGKB (Whirl-Carrillo et al., 2012; Barbarino et al., 2018), with the *in silico* drug prescriptions from the Cancer Genome Interpreter (Tamborero et al., 2018), and with the current clinical trials in advanced stages from the Open Targets Platform (Carvalho-Silva et al., 2019). The main idea of the integration of precision oncology per human population is to prioritize the possible oncogenic variants found in cancer patients, focusing economic resources for PGx testing in a most effective way.

**FIGURE 10:**
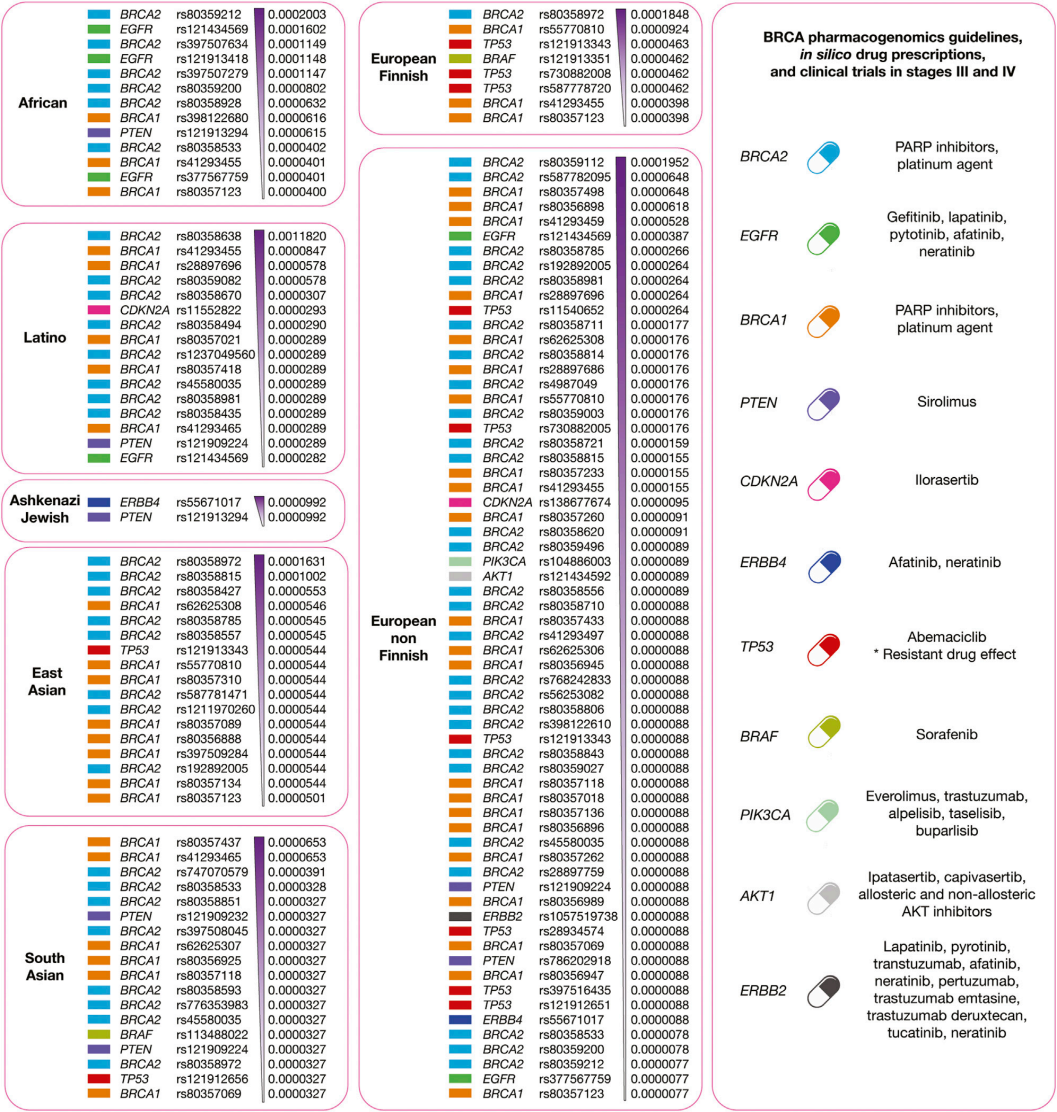
Precision medicine of BRCA per human population. On the left, druggable BRCA driver proteins carrying known oncogenic variants with the highest allele frequencies and the highest deleteriousness scores per human population. On the right, integration of pharmacogenomics guidelines, drug prescription, and clinical trials according to druggable BRCA driver proteins.

Figure 10 summarizes the integration of precision oncology of BRCA per human population. There are 11 druggable driver proteins carrying 138 known oncogenic variants with the highest deleteriousness scores, and the highest allele frequencies per human population. Latinos have 16 variants in five actionable therapeutic targets (BRCA1, BRCA2, CDKN2A, PTEN, and EGFR); Africans have 13 variants in four druggable proteins (BRCA1, BRCA2, EGFR, and PTEN); Ashkenazi Jewish has 2 variants in 2 actionable therapeutic targets (ERBB4 and PTEN); East Asians have 17 variants in 3 druggable proteins (BRCA1, BRCA2, and TP53); European Finnish have eight variants in four actionable therapeutic targets (BRCA1, BRCA2, TP53, and BRAF); European non-Finnish have 64 variants in 10 druggable proteins (BRCA1, BRCA2, EGFR, TP53, CDKN2A, PIK3CA, AKT1, PTEN, ERBB2, and ERBB4); and South Asians have 18 variants in five actionable therapeutic targets (BRCA1, BRCA2, PTEN, BRAF, and TP53) (Supplementary Table S14). Regarding BRCA responsive treatments, ipatasertib, capivasertib, allosteric AKT inhibitors, and non-allosteric AKT inhibitors act on AKT (Kostaras et al., 2020); veliparib and cisplatin respond on BRCA1 and BRCA2 (Diéras et al., 2020); ilorasertib reacts on CDKN2A (Aftab et al., 2019); sirolimus acts on PTEN (Schmid et al., 2014); gefitinib, lapatinib, pytotinib, afatinib, and neratinib respond on EGFR; afatinib and neratinib react on ERBB4; sorafenib acts on BRAF; everolimus, trastuzumab, alpelisib, taselisib, and buparlisib work on PIK3CA (Chen et al., 2019); lastly, lapatinib, pyrotinib, transtuzumab, afatinib, neratinib, trastuzumab emtasine, pertuzumab, trastuzumab deruxtecan, tucatinib, and neratinib act on ERBB2 (Ben-Baruch et al., 2015).

**FIGURE 11:**
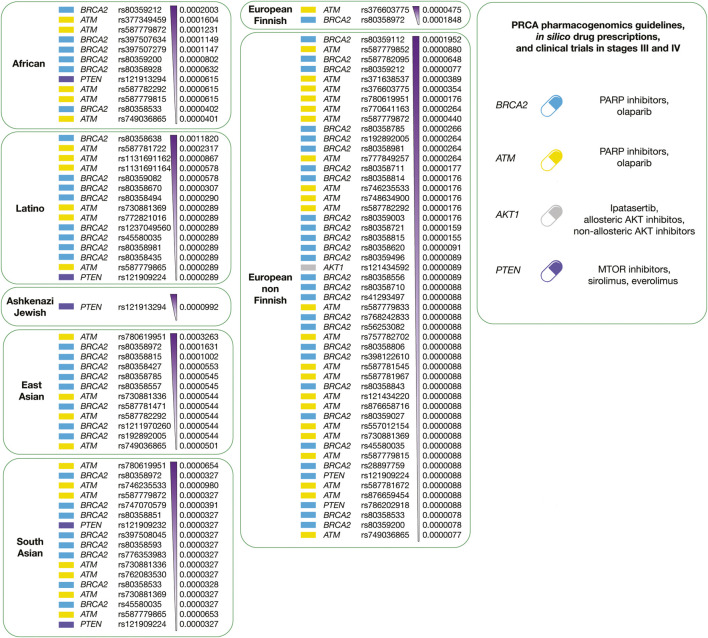
Precision medicine of PRCA per human population. On the left, druggable PRCA driver proteins carrying known oncogenic variants with the highest allele frequencies and the highest deleteriousness scores per human population. On the right, integration of pharmacogenomics guidelines, drug prescription, and clinical trials according to druggable PRCA driver proteins.

Figure 11 summarizes the integration of precision oncology of PRCA per human population. There are four druggable driver proteins carrying 110 known oncogenic variants with the highest deleteriousness scores, and the highest allele frequencies per human population. Latinos have 15 variants in 3 actionable therapeutic targets (BRCA2, ATM, and PTEN); Africans have 12 variants in 3 druggable proteins (BRCA2, ATM, and PTEN); Ashkenazi Jewish has 1 variant in 1 actionable therapeutic target (PTEN); East Asians have 12 variants in 2 druggable proteins (BRCA2 and ATM); European Finnish have 2 variants in 2 actionable therapeutic targets (BRCA2 and ATM); European non-Finnish have 51 variants in four druggable proteins (BRCA2, AKT1, ATM, and PTEN); and South Asians have 17 variants in 3 actionable therapeutic targets (BRCA2, ATM, and PTEN) (Supplementary Table S15). Regarding PRCA responsive treatments, ipatasertib, allosteric and non-allosteric AKT inhibitors act on AKT1 (Mundi et al., 2016); olaparib (PARP inhibitor) reacts on ATM and BRCA2 (de Bono et al., 2020); and lastly, sirolimus and everolimus (MTOR inhibitors) act on PTEN (Morgan et al., 2009).

In the era of precision oncology, PGx testing will make it possible to improve the efficiency on the use of resources, patient safety, and drug dosage in BRCA and PRCA treatments. Hence, it is imperative to unify efforts where developing countries might invest in obtaining databases of their population’s genomic profiles, and developed countries might incorporate racial/ethnic minority populations in future clinical trials and cancer researches with the main aim of fomenting PGx in public health policies and clinical practice.

## Data Availability

The original contributions presented in the study are included in the article/Supplementary Material, further inquiries can be directed to the corresponding authors.The datasets generated for this study are included in this published article (and its Supplementary Information files).
